# A retrospective longitudinal cohort study of the clinical burden in myasthenia gravis

**DOI:** 10.1186/s12883-022-02692-4

**Published:** 2022-05-09

**Authors:** Linda Harris, Sophie Graham, Sharon MacLachlan, Alex Exuzides, Saiju Jacob

**Affiliations:** 1 Biohaven Pharmaceuticals, 215 Church Street, New Haven, CT 06510 USA; 2 The Ark, 201 Talgarth Road, London, W6 8DL UK; 3 450 Sansome Street, Suite 650, San Francisco, CA 94111 USA; 4 Department of Neurology and Centre for Rare Diseases, Institute of Immunology and Imunotherapy, University Hospitals Birmingham and University of Birmingham, Mindelsohn Way, Edgbaston, Birmingham, B15 2GW UK

**Keywords:** Myasthenia gravis, Refractory, Burden of illness, England, Myasthenic crisis, GPRD

## Abstract

**Background:**

Patients with generalized myasthenia gravis (MG) often experience debilitating exacerbations, with the possibility of life-threatening respiratory crises requiring hospitalization. Long-term longitudinal studies are needed to understand the burden of MG, including in patients whose disease is refractory to conventional treatment.

**Methods:**

A retrospective, longitudinal, cohort study was conducted of patients in England aged ≥ 18 years with treatment-refractory or non-refractory MG, using data recorded during 1997–2016 in the Clinical Practice Research Datalink and the Hospital Episode Statistics databases. A control cohort of patients without MG, matched to the patients in the treatment-refractory MG cohort, was also identified. Outcome measures included myasthenic crises, MG exacerbations, MG-related hospitalizations, comorbidities, and all-cause mortality. Descriptive statistics were calculated for the overall MG population. For continuous variables, between-cohort comparisons were made using *t* tests for normally distributed data and Mann–Whitney *U* tests for non-normally distributed data. For categorical data, the comparisons were made by chi-squared tests. Differences in clinical outcomes between cohorts were modeled using negative binomial regression.

**Results:**

A total of 1149 patients with MG were included. Overall, 18.4% of patients experienced myasthenic crises, 24.6% experienced exacerbations, and 38.6% underwent MG-related hospitalizations. Most of these events occurred within 2–3 years of diagnosis. Patients with MG refractory to conventional treatment (*n* = 66) experienced more exacerbations and MG-related hospitalizations than patients with non-refractory disease (*n* = 1083). Patients with refractory MG experienced a higher frequency of renal disease and hypertension compared with patients with non-refractory MG, and with matched patients without MG. They were also more likely to have diabetes and congestive heart failure than the matched controls. Rates of all-cause mortality during the follow-up period did not differ between patients with refractory MG and non-refractory MG.

**Conclusions:**

These results show that conventional treatments for MG are not adequately managing patients’ symptoms and that patients with refractory MG are more likely to experience certain comorbidities than those with non-refractory MG or matched controls without MG. Future research should focus on the impact of newer targeted therapies on long-term clinical outcomes and comorbid conditions.

**Supplementary Information:**

The online version contains supplementary material available at 10.1186/s12883-022-02692-4.

## Introduction

Myasthenia gravis (MG) is an autoimmune neuromuscular disease that causes weakness of skeletal muscles, usually first manifesting as droopy eyelids and double vision [1, 2]. In most cases, it progresses to bulbar and limb weakness [3, 4], which can cause difficulties performing daily tasks [2]. Patients with generalized MG [gMG] often experience debilitating exacerbations, with the possibility of life-threatening respiratory crises requiring intubation and mechanical ventilation [5]. Complications of MG crisis include fever, respiratory infections, atelectasis, arrhythmias, heart failure, and hypotension [6]. Adding to this burden, patients with gMG often develop comorbidities, such as cardiovascular disease, hyperlipidemia, hypertension, diabetes mellitus, respiratory disorders, and concomitant autoimmune diseases [7, 8], all of which can lengthen hospital stays and increase the risk of death [9]. The burden of MG can be further worsened by the adverse effects of medications; for example, prolonged corticosteroid use can cause osteoporosis, weight gain, cardiac conditions, gastrointestinal conditions, hypertension, glucose intolerance, and diabetes [8, 10, 11].

Long-term studies on MG are critical to understanding the burden of disease and the effects of treatments. Studies published to date provide important data on MG but were carried out at least two decades ago and may not fully represent the current burden of disease in patients with MG. Two studies at a US hospital during the 1950s [12] and 1960–1980 [13] found that within the first 2 years of diagnosis, 13.7–17.3% of patients with MG experienced a myasthenic crisis. The largest longitudinal study on MG, conducted in the US during 1940–2000, found that after MG manifested, it rapidly progressed to generalized weakness in 80% of patients [3]. The study also found that although most patients improved after the first 2 years, patients who worsened were less likely to survive.

An estimated 5–15% of patients have MG considered to be refractory to conventional treatment and experience a greater clinical and treatment burden than patients with non-refractory disease [14–18]. Studies of insurance claims data in the US and Japan indicated that, during the first year after a diagnosis of MG was recorded, patients with refractory MG had a greater burden of MG and associated healthcare resource utilization, including the need for hospitalization and emergency room visits, than patients with non-refractory MG [14–16]; however, limited data are available on the long-term burden of refractory MG.

We recently described healthcare resource utilization by patients with refractory and non-refractory MG in England using data from the Clinical Practice Research Datalink (CPRD) and Hospital Episode Statistics (HES) databases collected from 1997 to 2016 [19]. As reported in the US and Japan using claims data [14, 16], the rates of general practitioner visits, visits to other healthcare professionals, outpatient visits, and inpatient hospitalization were significantly higher for patients with refractory MG than for patients with non-refractory MG. Here, using the same dataset, we assessed the characteristics, comorbidities, and clinical burden in the overall MG population in England and examined how these differ between patients with refractory and non-refractory MG. Such data will provide valuable insight into the course, management, and impact of this rare disease.

## Methods

### Study design and conduct

This was a retrospective, longitudinal, observational cohort study using linked data from CPRD and HES between April 1, 1997, and December 31, 2016. Details of the study design were described previously [19]. Briefly, the study included patients in England with a diagnosis of MG who were ≥ 18 years of age at the date of first MG diagnosis (index date) and who had linked data in CPRD and HES. No exclusion criteria were applied. Data extracted included diagnoses and associated dates, demographics at the index date (age, sex, and ethnicity), types and dates of treatments and procedures, dates of inpatient hospitalizations, comorbidities included in the Charlson Comorbidity Index [20], autoimmune comorbidities, and hypertension [15]. The Charlson Comorbidity Index score was calculated using the validated weights described by Quan et al. [21]. Deaths were identified from CPRD or Office for National Statistics records, with the date taken as the earlier reported in the two databases.

Outcome measures included myasthenic crises, MG exacerbations, MG-related hospitalizations, and all-cause mortality. Myasthenic crisis was defined as respiratory distress, respiratory failure, respiratory support, intubation, or mechanical ventilation (see Supplementary Table 1, Additional File 1 for diagnosis codes). MG exacerbations included events specifically coded as MG exacerbations, myasthenic crises, intravenous immunoglobulin administration, and plasmapheresis (see Supplementary Table 2, Additional File 1 for diagnosis codes). MG-related hospitalizations were defined as any hospitalization with MG as the primary admission diagnosis (see Supplementary Table 3, Additional File 1 for diagnosis codes).

The baseline period spanned the up-to-standard date (date at which the general practice had continuous and complete recording of data), current registration date, or start of the study period, whichever occurred last, up to the index date. The follow-up period included the day after the index date up until the patient transferred out of the practice, the last date of data collection, or the study end, whichever occurred first.

As described in our previous analysis of healthcare utilization in patients with MG [19], patients were classified as having refractory or non-refractory disease using the 2016 Consensus guidelines definition of refractory (“post-Intervention Status is unchanged or worse after corticosteroids and at least 2 other IS agents, used in adequate doses for an adequate duration, with persistent symptoms or side effects that limit functioning, as defined by patient and physician” [22]), the Mantegazza et al. [23] definition of refractory (“inability to reduce immunosuppressive therapy without clinical relapse or a need for ongoing rescue therapy such as intravenous immunoglobulin G (IVIg) or plasma exchange”) and an algorithm that was adapted from a study of US claims data [15], which was altered to reflect UK clinical treatment guidelines for gMG [24] and to fit the data available in the CPRD and HES. Briefly, to be classified as refractory, patients with MG had to: (1) have been referred to a neurologist and (2) meet one of the following criteria: (a)(i) ≥ 2 different immunosuppressive therapies prescribed (azathioprine, mycophenolate mofetil, methotrexate, ciclosporin, tacrolimus, or cyclophosphamide) after the index date or (ii) > 3 treatment episodes of the same immunosuppressive therapies within 24 months of the index date; or (b) ≥ 1 immunosuppressive therapy prescribed any time after the index date and ≥ 4 hospital treatments (plasmapheresis or intravenous immunoglobulins) ≥ 2 months apart within a year of the index date. Criterion 2ai aims to identify patients that do not respond to, or experience an adverse event when on a specific immunosuppressive therapy (i.e., 2016 Consensus guidelines: “side effects that limit functioning”). Criterion 2aii aims to identify patients that cannot reduce their immunosuppressive therapy without clinical relapse (i.e., Mantegazza et al. definition of refractory). For criterion 2aii, a treatment episode was when a patient had consecutive prescriptions of the same immunosuppressive therapy within 90 days of the previous. If there was then no prescription within a 90-day period of the last prescription within this continuous treatment episode, then the patient was considered to have discontinued treatment. Patients that then subsequently restarted the same treatment were considered to have another treatment episode. This needed to be discontinued again and then restarted in order for patients to meet the criteria for this part of the algorithm (i.e., 3 treatment episodes). All patients with MG not identified as having refractory disease using this algorithm were considered to have non-refractory disease.

A non-MG control cohort of patients with linked CPRD-HES data was randomly matched (4:1) to patients in the refractory cohort by age, sex, and general practice. To ensure that the control patients did not have a diagnosis of MG, they had to have ≥ 12 months of observation between the up-to-standard date and the matched reference date.

### Data sources

The CPRD is one of the largest sets of routinely collected longitudinal electronic medical records. It is considered generally representative of the UK population and contains high-quality longitudinal data from general practices across the UK [25]. At the time this study was conducted, the CPRD included 717 practices, representing approximately 8% of the UK population. The HES database includes all inpatient admissions in England as well as outpatient specialist and emergency room visits [26]. The CPRD is linked to HES for approximately 75% of general practices contributing to the CPRD in England, and it is this subset that is included in our study.

### Statistical analyses

For the overall MG population, only descriptive statistics were calculated. For continuous variables, comparisons between refractory and non-refractory MG cohorts and between refractory MG and non-MG control cohorts were made by *t* tests for normally distributed data and Mann–Whitney *U* tests for non-normally distributed data. For categorical data, comparisons between cohorts were made by chi-squared tests. Differences in clinical outcomes between cohorts were modeled using negative binomial regression. Separate models were run to assess the impact of refractory MG vs non-refractory MG on the number of MG crises, exacerbations, and MG-related inpatient hospitalizations during the follow-up period. Baseline covariates included age, sex, Charlson Comorbidity Index score, and pre-specified comorbidities. Follow-up time was included as an offset variable to control for varying follow-up time for each patient. Statistical significance was assumed for *p*-values < 0.05. Statistical analyses were performed using SAS (version 9.4; SAS Institute Inc., Cary, NC, USA).

## Results

This study included 1149 patients with MG, of whom 66 (5.7%) were classified as having refractory disease, as described previously [19]. The median baseline period for the full population was 88.2 (interquartile range, 36.5–152.5) months, and the median follow-up period was 47.2 (interquartile range, 19.7–90.3) months.

### Overall population of patients with MG

#### Demographics

On the index date, the mean age (standard deviation [SD]) in the full MG population was 63.6 (16.7) years, and approximately two-thirds (67.2%) were ≥ 60 years of age (Table 1). Just over half (53.0%) of the patients were male, and most (86.2%) were White. All geographic regions of England were well represented.

**Table 1:** Demographics of the study population with myasthenia gravis (*N* = 1149)

Characteristic	Value
Age (y), mean ± standard deviation	63.6 ± 16.7
Age category (y), n (%)	
18–39	136 (11.8)
40–59	241 (21.0)
60–79	609 (53.0)
≥ 80	163 (14.2)
Sex, n (%)	
Male	609 (53.0)
Female	540 (47.0)
Geographic region, n (%)	
North East	21 (1.8)
South East	149 (13.0)
North West	165 (14.4)
Yorkshire & The Humber	47 (4.1)
East Midlands	26 (2.3)
West Midlands	123 (10.7)
East of England	144 (12.5)
South West	162 (14.1)
South Central	129 (11.2)
London	183 (15.9)
Ethnicity, n (%)	
Asian	32 (2.8)
Black	17 (1.5)
Other	105 (9.1)
Mixed	5 (0.4)
White	990 (86.2)
Charlson Comorbidity Index score, mean ± standard deviation	0.7 ± 1.5
Charlson Comorbidity Index category, n (%)	
0 to < 1	950 (82.7)
1 to < 2	112 (9.7)
2 to < 3	40 (3.5)
3 to < 4	16 (1.4)
≥ 4	31 (2.7)

#### Comorbidities

At the index date, most patients (87.2%) had a Charlson Comorbidity Index score < 1.0 (Table 1). The most common comorbidities (≥ 10%) during the baseline period were hypertension (38.0%), diabetes without chronic complications (13.4%), ankylosing spondylitis (12.6%), chronic pulmonary disease (11.2%), and renal disease (11.1%) (Table 2). During the follow-up period, the most common were renal disease (23.1%), chronic pulmonary disease (20.5%), diabetes without complications (20.1%), malignancies (16.8%), hypertension (15.0%), myocardial infarction (14.7%), and congestive heart failure (13.2%).

**Table 2:** Comorbidities in the study population with myasthenia gravis (*N* = 1149)

Comorbidity	No. of patients (%)	
	**Baseline (pre-index to index) period**	**Follow-up (post-index) period**
Myocardial infarction	83 (7.2)	169 (14.7)
Congestive heart failure	44 (3.8)	152 (13.2)
Peripheral vascular disease	61 (5.3)	79 (6.9)
Cerebrovascular disease	106 (9.2)	92 (8.0)
Dementia	13 (1.1)	70 (6.1)
Chronic pulmonary disease	129 (11.2)	236 (20.5)
Rheumatologic disease	37 (3.2)	58 (5.0)
Peptic ulcer disease	50 (4.4)	9 (0.8)
Mild liver disease	16 (1.4)	37 (3.2)
Moderate or severe liver disease	3 (0.3)	13 (1.1)
Diabetes without chronic complications	154 (13.4)	231 (20.1)
Diabetes with chronic complications	43 (3.7)	56 (4.9)
Hemiplegia or paraplegia	14 (1.2)	22 (1.9)
Renal disease	128 (11.1)	265 (23.1)
Malignancy	99 (8.6)	193 (16.8)
Metastatic solid tumor	14 (1.2)	62 (5.4)
HIV/AIDS	0 (0.0)	1 (0.1)
Hypertension	437 (38.0)	172 (15.0)
Ankylosing spondylitis	145 (12.6)	88 (7.7)
Psoriasis	50 (4.4)	25 (2.2)
Psoriatic arthritis	3 (0.3)	6 (0.5)
Crohn’s disease	7 (0.6)	4 (0.3)
Ulcerative colitis	10 (0.9)	12 (1.0)
Systemic lupus erythematosus	7 (0.6)	5 (0.4)

*AIDS* acquired immune deficiency syndrome; *HIV* human immunodeficiency virus

#### Treatments

During the full follow-up period, patients received a median of two different treatments for MG. The most frequently prescribed were pyridostigmine (70.3% of patients), prednisolone (61.6%), and azathioprine (24.9%) (Table 3). Intravenous immunoglobulin was administered to 9.6% of patients, with a median number of one treatment per treated patient. Plasmapheresis was administered to 2.1% of patients, with a median number of one treatment per treated patient.

**Table 3:** Prescriptions during the follow-up period in the overall myasthenia gravis population (*N* = 1149)

Treatment^a^	No. of patients (%)	Median No. of prescriptions per patient (interquartile range)
Pyridostigmine	808 (70.3)	13 (5–34.5)
Prednisolone	708 (61.6)	15.5 (5–37.5)
Azathioprine	286 (24.9)	29 (7–66)
Mycophenolate mofetil	40 (3.5)	20.5 (11–52)
Methotrexate	47 (4.1)	16 (6–52)
Ciclosporin	2 (0.2)	26 (12–40)
Tacrolimus	3 (0.3)	2 (1–814)
Cyclophosphamide	1 (0.1)	3 (3–3)
Plasmapheresis	24 (2.1)	1 (1–1)
Intravenous immunoglobulin	110 (9.6)	1 (1–2)

^a^Categories are not exclusive

#### MG events during the follow-up period

Myasthenic crises were experienced by 18.4% (211/1149) of patients, MG exacerbations (which included crises and other exacerbations) by 24.6% (283/1149), and MG-related inpatient hospitalizations by 38.6% (444/1149) (Table 4). The patients experiencing these events had a mean of 1.4 myasthenic crises (median, 0.37), 2.8 exacerbations (median, 0.44), and 2.2 MG-related hospitalizations (median, 0.45) per year.

**Table 4:** MG crises, exacerbations, and hospitalizations during the follow-up period (*N* = 1149)

Event	No. of patients (%)	Events per year in affected patients	
		**Mean** ± **standard deviation**	**Median (interquartile range)**
Myasthenic crisis	211 (18.4)	1.4 ± 4.3	0.37 (0.18–0.84)
MG exacerbation	283 (24.6)	2.8 ± 10.3	0.44 (0.20–1.27)
MG-related inpatient hospitalization	444 (38.6)	2.2 ± 9.1	0.45 (0.21–1.18)

*MG* myasthenia gravis

### Comparison of refractory and non-refractory MG

Baseline characteristics according to refractory status have been described previously and were similar in the refractory and non-refractory MG cohorts [19].

#### Clinical burden

Of patients who experienced exacerbations, those with refractory MG experienced statistically significantly more exacerbations during the 10 years after the index date than patients with non-refractory MG [mean (SD) 8.71 (15.21) vs 3.09 (9.04), respectively; *p* = 0.0009]. This was also the case for the number of MG-related hospitalizations [5.00 (11.08) vs 1.79 (1.42); *p* = 0.0011] but not for the number of myasthenic crises [2.00 (2.27) vs 1.71 (1.77); *p* = 0.97]. Results were similar when accounting for the full follow-up period and adjusted for baseline characteristics: patients with refractory MG experienced statistically significantly more exacerbations (*p* = 0.0022) and MG-related hospitalizations (*p* = 0.0013) but not myasthenic crises (*p* = 0.48) than patients with non-refractory MG (Table 5).

**Table 5:** Differences in MG crises, exacerbations, and hospitalizations between patients with refractory and non-refractory disease

Event	Percent difference in number of events between refractory and non-refractory MG (95% confidence interval)^a^	*P*-value^b^
Myasthenic crisis	− 27.9 (− 71.1, 79.1)	0.4816
MG exacerbation	309.3 (66.1, 908.8)	0.0022
MG-related inpatient hospitalization	73.0 (23.8, 141.8)	0.0013

*MG* myasthenia gravis

^a^Calculated as events in patients with refractory disease minus events in those with non-refractory disease. ^b^Numbers of events during the follow-up period were compared by negative binomial regression with age, sex, Charlson Comorbidity Index, and pre-specified comorbidities as baseline covariates

**Fig. 1:**
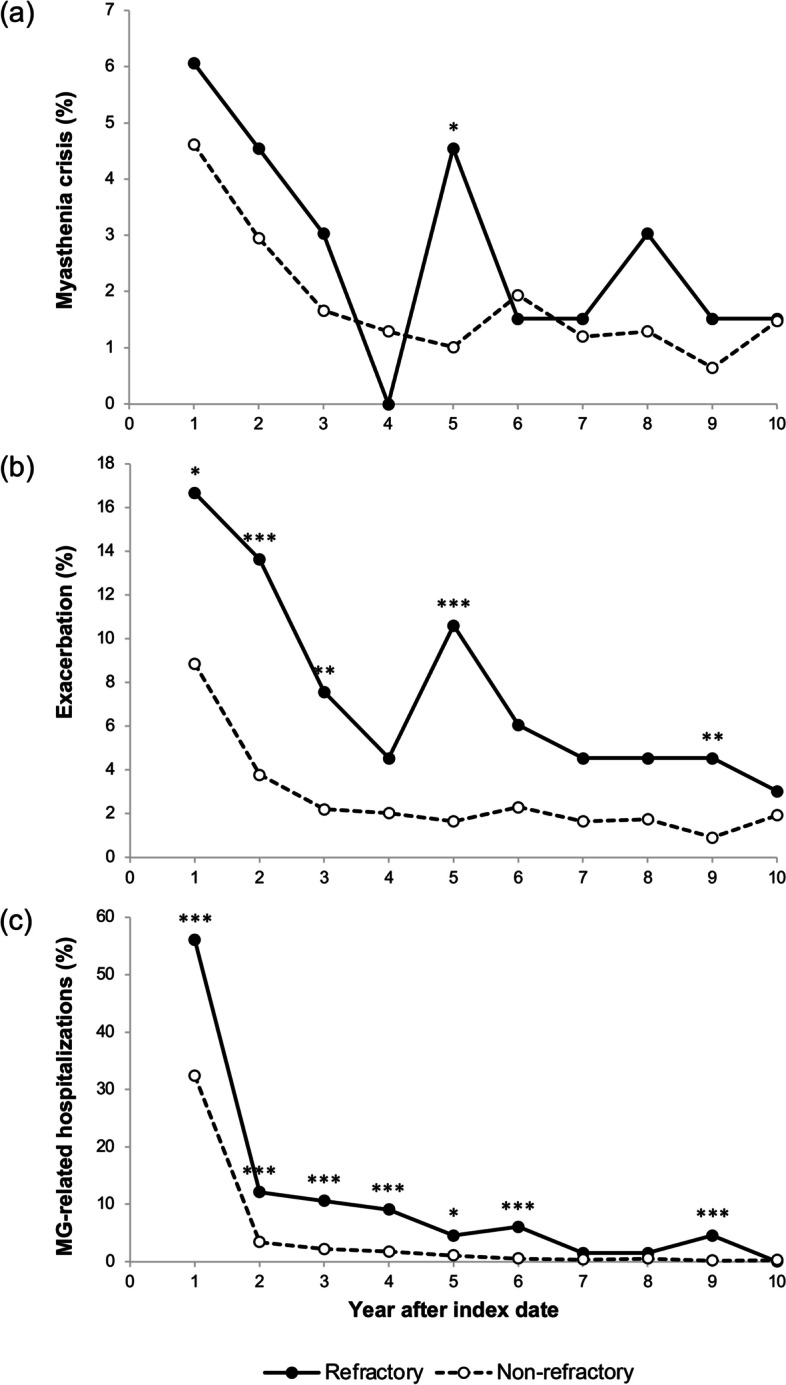
Proportions of patients with refractory and non-refractory MG who experienced MG-related events. Annual incidence of MG crises **a**, exacerbations **b**, and inpatient hospitalizations **c** following the index date. MG myasthenia gravis. Statistical difference for refractory vs non-refractory MG: *p < 0.05; **p < 0.01; ***p < 0.001

Proportions of patients with myasthenia crises, exacerbations, and MG-related hospitalizations were highest the first year after the index date and decreased progressively in both the refractory and non-refractory MG cohorts (Fig. 1). During the first 6 years after the first diagnosis of MG, the proportion of patients experiencing MG-related hospitalizations each year was higher in the refractory cohort than in the non-refractory cohort. The proportion of patients experiencing exacerbations was higher in the refractory cohort than in the non-refractory cohort, although differences were largest during the first few years after diagnosis. The proportion of patients experiencing myasthenia crises each year appeared to be slightly higher in the refractory than in the non-refractory cohort.

#### Comorbidities

During the baseline period, comorbidities were similar in patients with refractory and non-refractory MG, except for psoriatic arthritis, which was more common in patients with refractory than non-refractory MG (Table 6). During the follow-up period, comorbidities more frequently reported in patients with refractory than non-refractory MG included renal disease (33.3% [22/66] vs 22.4% [243/1083], respectively), and hypertension (24.2% [16/66] vs 14.4% 156/1083]). These same comorbidities, along with diabetes with or without complications, and congestive heart failure were also significantly more common in the refractory cohort than the age- and sex-matched controls during follow up (Table 6).

**Table 6:** Comorbidities in patients with refractory and non-refractory MG and age- and sex-matched controls

	**Baseline (pre-index to index) period**					**Follow-up (post-index) period**				
	**Refractory MG**	**Non-refractory MG**	**Refractory vs non-refractory**	**Controls**^**a**^	**Refractory MG vs controls**	**Refractory MG**	**Non-refractory MG**	**Refractory vs non-refractory**	**Controls**^**a**^	**Refractory MG vs controls**
	**(*N* = 66)**	**(*N* = 1083)**	***P*-value**	**(*N* = 252)**	***P*-value**	**(*N* = 66)**	**(*N* = 1083)**	***P*-value**	**(*N* = 252)**	***P*-value**
**Comorbidity**	**n (%)**	**n (%)**		**n (%)**		**n (%)**	**n (%)**		**n (%)**	
Myocardial infarction	3 (4.6)	80 (7.4)	0.39	10 (4.0)	0.83	9 (13.6)	160 (14.8)	0.80	21 (8.3)	0.19
Congestive heart failure	1 (1.5)	43 (4.0)	0.31	8 (3.2)	0.47	13 (19.7)	139 (12.8)	0.11	25 (9.9)	0.03
Peripheral vascular disease	3 (4.6)	58 (5.4)	0.78	6 (2.4)	0.35	5 (7.6)	74 (6.8)	0.82	14 (5.6)	0.54
Cerebrovascular disease	2 (3.0)	104 (9.6)	0.07	12 (4.8)	0.54	5 (7.6)	87 (8.0)	0.89	20 (7.9)	0.92
Dementia	0 (0.0)	13 (1.2)	0.37	2 (0.8)	0.47	5 (7.6)	65 (6.0)	0.60	15 (6.0)	0.63
COPD	6 (9.1)	123 (11.4)	0.57	16 (6.4)	0.43	13 (19.7)	223 (20.6)	0.86	35 (13.9)	0.24
Rheumatologic disease	3 (4.6)	34 (3.1)	0.53	3 (1.2)	0.07	5 (7.6)	53 (4.9)	0.33	9 (3.6)	0.16
Peptic ulcer disease	2 (3.0)	48 (4.4)	0.59	5 (2.0)	0.61	0 (0.0)	9 (0.8)	0.46	4 (1.6)	0.30
Liver disease, mild	2 (3.0)	14 (1.3)	0.24	0 (0.0)	< 0.01	4 (6.1)	33 (3.1)	0.18	7 (2.8)	0.19
Liver disease, moderate–severe	0 (0.0)	3 (0.3)	0.67	0 (0.0)	NC	1 (1.5)	12 (1.1)	0.76	3 (1.2)	0.83
Hemiplegia or paraplegia	1 (1.5)	13 (1.2)	0.82	1 (0.4)	0.31	1 (1.5)	21 (1.9)	0.81	3 (1.2)	0.83
Renal disease	8 (12.1)	120 (11.1)	0.79	20 (7.9)	0.29	22 (33.3)	243 (22.4)	0.04	51 (20.2)	0.02
Malignancy	6 (9.1)	93 (8.6)	0.89	22 (8.7)	0.93	14 (21.2)	179 (16.5)	0.32	41 (16.3)	0.34
Metastatic solid tumor	1 (1.5)	13 (1.2)	0.82	4 (1.6)	0.97	3 (4.6)	59 (5.5)	0.75	14 (5.6)	0.75
HIV/AIDS	0 (0.0)	0 (0.0)	NC	0 (0.0)	NC	0 (0.0)	1 (0.1)	0.80	0 (0.0)	NC
Hypertension	22 (33.3)	415 (38.3)	0.42	71 (28.2)	0.41	16 (24.2)	156 (14.4)	0.03	32 (12.7)	0.02
Ankylosing spondylitis	9 (13.6)	136 (12.6)	0.80	27 (10.7)	0.50	5 (7.6)	83 (7.7)	0.98	12 (4.8)	0.37
Psoriasis	4 (6.1)	46 (4.3)	0.48	4 (1.6)	0.04	4 (6.1)	21 (1.9)	0.03	3 (1.2)	0.02
Psoriatic arthritis	2 (3.0)	1 (0.1)	< 0.0001	0 (0.0)	< 0.01	2 (3.0)	4 (0.4)	< 0.01	0 (0.0)	< 0.01
Crohn's disease	0 (0.0)	7 (0.7)	0.51	1 (0.4)	0.61	0 (0.0)	4 (0.4)	0.62	1 (0.4)	0.61
Ulcerative colitis	0 (0.0)	10 (0.9)	0.43	0 (0.0)	NC	0 (0.0)	12 (1.1)	0.39	0 (0.0)	NC
Systemic lupus erythematosus	1 (1.5)	6 (0.6)	0.33	1 (0.4)	0.31	1 (1.5)	4 (0.4)	0.17	0 (0.0)	0.05
Lupus nephritis	0 (0.0)	0 (0.0)	NC	0 (0.0)	NC	0 (0.0)	0 (0.0)	NC	0 (0.0)	NC
Diabetes without chronic complications	8 (12.1)	146 (13.5)	0.75	23 (9.1)	0.47	18 (27.3)	213 (19.7)	0.13	35 (13.9)	0.01
Diabetes with chronic complications	3 (4.6)	40 (3.7)	0.72	6 (2.4)	0.35	6 (9.1)	50 (4.6)	0.10	5 (2.0)	< 0.01

*AIDS* acquired immune deficiency syndrome, *COPD* chronic obstructive pulmonary disease, *HIV* human immunodeficiency virus, *MG* myasthenia gravis, *NC* not calculated

^a^ The non-MG control cohort was randomly matched 4:1 to patients in the refractory cohort by age, sex, and general practice. To ensure that the control patients did not have a diagnosis of MG, they had to have at least 12 months of observation between the up-to-standard date and the matched reference date

#### Mortality

Rates of all-cause mortality during the follow-up period did not differ between patients with refractory MG (15.2% [10/66]) and non-refractory MG (23.3% [252/1083]; *p* = 0.13) or between patients with refractory MG and non-MG controls (11.5% [29/252]; *p* = 0.42). Age at death did not differ between patients with refractory MG and non-refractory MG [mean SD) 74.4 (9.7) vs 78.6 (10.2) years, respectively; *p* = 0.15].

## Discussion

The current study assessed the clinical burden of MG in a representative population of over 1000 patients in England over two decades using linked data from the CPRD and HES. The study showed that patients diagnosed with MG often experience severe MG-related events, with 39% being hospitalized at least once for MG, 25% experiencing at least one exacerbation, and 18% experiencing at least one myasthenic crisis. Many of these events occurred within the first 2–3 years after MG was diagnosed, indicating that in most cases, the disease is ultimately controlled by treatment, spontaneously subsides, or both. We also found that for several years after diagnosis—even beyond the first 2–3 years—patients who were refractory to conventional treatment continued to experience more exacerbations and MG-related hospitalizations than patients with non-refractory disease. More frequent comorbidities, some of which may have been due to treatments, especially long-term corticosteroid use, further added to the burden of refractory MG.

Few other longitudinal studies have examined how the burden of MG changes over time, and all were completed several decades ago [3, 12, 13]. In addition, sample sizes were modest in most of these studies, except for a study by Grob et al., which included 1976 patients with MG in the US over the six decades from 1940 to 2000 [3]. Two recent longitudinal studies of claims data, one in the US [15] and the other in Japan [16], focused on the burden of illness in patients with refractory and non-refractory MG, although analyses were limited to the first year after diagnosis. In line with the current study and our previous analysis of healthcare resource utilization in this same population [19], the US and Japanese claims studies showed more frequent exacerbations, hospitalizations, and other healthcare resource utilization in patients with refractory vs non-refractory MG. Unlike the US and Japanese claims studies, however, the current study did not find a difference in the proportion of patients experiencing myasthenic crises. This might be related to differences in definitions of myasthenic crisis or refractory status, although insufficient numbers of patients with refractory disease may have precluded making inferences. Also, in the current study, patients did not meet the definition of refractory MG if they died within the first 2 years after diagnosis, which is when most myasthenic crises occur.

Comorbidities contribute to the burden of MG, lengthen hospital stays, and increase the risk of death. In patients with MG, reported comorbidities include cardiovascular disease, hyperlipidemia, hypertension, diabetes mellitus, respiratory disorders, and concomitant autoimmune diseases [7, 8]. The current study provides data on comorbidities before and after MG was diagnosed. The most common comorbidities seen during the baseline and follow-up periods were renal disease, chronic pulmonary disease, diabetes, malignancies, hypertension, myocardial infarction, and congestive heart failure. These are all common in older age, and as expected in this population, which was mostly > 60 years of age. However, renal disease, hypertension, psoriasis, and psoriatic arthritis were more common in patients with refractory than non-refractory MG. Diabetes, renal disease, hypertension, congestive heart failure, psoriasis, and psoriatic arthritis were more frequent in patients with refractory MG than in age- and sex-matched non-MG controls. The study of US claims data also reported more frequent diabetes, cardiac arrhythmias, and severe infections in patients with refractory than non-refractory MG [15]. Some of the comorbidities associated with refractory MG are likely adverse effects of long-term treatment with systemic corticosteroids and other immunosuppressive therapies [10, 27]. Also, increased rates of psoriasis and psoriatic arthritis in patients with refractory MG are consistent with more frequent concomitant autoimmune conditions in these patients [7].

The current study assessed the burden of MG in patients receiving conventional therapies (e.g. corticosteroids, immunosuppressive therapies, intravenous immunoglobulins, plasmapheresis) in 1997–2016. Since then, a humanized monoclonal antibody (eculizumab) that inhibits terminal complement activation has been approved for treatment of refractory gMG, and other targeted therapies are being developed. This study was conducted before the introduction of these newer therapies and is expected to provide a reference point for other studies that will be conducted after the introduction of these therapies.

One limitation of this study is that it was performed using healthcare data from the English primary care setting and therefore might not be generalizable to other countries. Nonetheless, this study provides extensive and long-term longitudinal data about periods before and after a diagnosis of MG was recorded. The more than 1000 patients with MG identified in this study represent one of the larger sets described to date, although low numbers of patients with refractory MG made it difficult to make inferences in some cases, such as for myasthenic crises and certain comorbidities.

Another potential limitation of this study is that the accuracy of the results depended on the completeness and precision of encoding by general practices for the CPRD and by hospitals for the HES. Further, the definition of refractory MG was adapted from existing guidelines [1, 22, 28, 29] for the databases used and, as such, depended mostly on records of treatments prescribed by general practitioners. Treatments administered in hospitals or by specialists are not recorded in CPRD or HES, which may have reduced the number of patients who should have been considered refractory. Additionally, because the reasons for procedures (e.g. intravenous immunoglobulin administration) cannot be determined from the HES, the definitions of exacerbations and crises may have resulted in a slight overestimation of their occurrence. Including clinical criteria may have improved the accuracy of detecting refractory cases, but such information was not available in the databases used for this study.

## Conclusions

The results of this study extend previous findings by providing recent data on the burden of MG over a long-term follow-up period and emphasize that patients with MG, especially those who are treatment refractory, have a heavy burden of illness, including frequent severe events and comorbidities. The higher prevalence of comorbidities in patients with refractory MG than in those with non-refractory MG may be related to treatments received, especially prolonged corticosteroid use. Therefore, there is a need for therapies targeting the underlying mechanism of disease. Future research should focus on describing the use of newer targeted therapies and their short- and long-term impact on clinical outcomes and comorbidities.

## Supplementary Information

**Additional file 1.** 

## Data Availability

The research data cannot be made available since the data source, the Clinical Practice Research Datalink, requires that data it supplies must be deleted two years after extraction. The data cannot be requested from CPRD since ethics approval is required to access the data.

## Abbreviations

CPRD: Clinical Practice Research Datalink
gMG: Generalized myasthenia gravis
HES: Hospital Episode Statistics
MG: Myasthenia gravis
SD: Standard deviation

## References

[1] Drachman DB (2016). Myasthenia gravis. Semin Neurol.

[2] Jacob S (2018). Refractory myasthenia gravis – patient burden and the need for new therapeutic targets. Eur Neurol Rev.

[3] Grob D, Brunner N, Namba T, Pagala M (2008). Lifetime course of myasthenia gravis. Muscle Nerve.

[4] Wang L, Zhang Y, He M. Clinical predictors for the prognosis of myasthenia gravis. BMC Neurol. 2017;17 1:77. 10.1186/s12883-017-0857-7. 10.1186/s12883-017-0857-7 PMC5395963 28420327

[5] Wendell LC, Levine JM (2011). Myasthenic crisis. Neurohospitalist.

[6] Thomas CE, Mayer SA, Gungor Y, Swarup R, Webster EA, Chang I (1997). Myasthenic crisis: clinical features, mortality, complications, and risk factors for prolonged intubation. Neurology.

[7] Mao ZF, Yang LX, Mo XA, Qin C, Lai YR, He NY (2011). Frequency of autoimmune diseases in myasthenia gravis: a systematic review. Int J Neurosci.

[8] Gilhus NE, Nacu A, Andersen JB, Owe JF (2015). Myasthenia gravis and risks for comorbidity. Eur J Neurol.

[9] Liu C, Wang Q, Qiu Z, Lin J, Chen B, Li Y (2017). Analysis of mortality and related factors in 2195 adult myasthenia gravis patients in a 10-year follow-up study. Neurol India.

[10] Rice JB, White AG, Scarpati LM, Wan G, Nelson WW (2017). Long-term systemic corticosteroid exposure: A systematic literature review. Clin Ther.

[11] Sussman J, Farrugia ME, Maddison P, Hill M, Leite MI, Hilton-Jones D (2015). Myasthenia gravis: Association of British Neurologists' management guidelines. Pract Neurol.

[12] Osserman KE, Kornfeld P, Cohen E, Genkins G, Mendelow H, Goldberg H (1958). Studies in myasthenia gravis; review of two hundred eighty-two cases at the Mount Sinai Hospital, New York City. AMA Arch Intern Med.

[13] Cohen MS, Younger D (1981). Aspects of the natural history of myasthenia gravis: crisis and death. Ann N Y Acad Sci.

[14] Boscoe AN, Xin H, L'Italien GJ, Harris LA, Cutter GR (2019). Impact of refractory myasthenia gravis on health-related quality of life. J Clin Neuromuscul Dis.

[15] Engel-Nitz NM, Boscoe A, Wolbeck R, Johnson J, Silvestri NJ (2018). Burden of illness in patients with treatment refractory myasthenia gravis. Muscle Nerve.

[16] Murai H, Hasebe M, Murata T, Utsugisawa K. Clinical burden and healthcare resource utilization associated with myasthenia gravis: Assessments from a Japanese claims database. Clin Exp Neuroimmunol. 2019;21:1–8.

[17] Sudulagunta SR, Sepehrar M, Sodalagunta MB, Settikere Nataraju A, Bangalore Raja SK, Sathyanarayana D, et al. Refractory myasthenia gravis - clinical profile, comorbidities and response to rituximab. Ger Med Sci. 2016;14:Doc12. 10.3205/000239. 10.3205/000239 PMC5067337 27790079

[18] Suh J, Goldstein JM, Nowak RJ (2013). Clinical characteristics of refractory myasthenia gravis patients. Yale J Biol Med.

[19] Harris L, Graham S, MacLachlan S, Exuzides A, Jacob S. Healthcare resource utilization by patients with treatment-refractory myasthenia gravis in England. J Med Econ. 2019:1–7. 10.1080/13696998.2019.1592180. 10.1080/13696998.2019.1592180 30841772

[20] Quan H, Sundararajan V, Halfon P, Fong A, Burnand B, Luthi JC (2005). Coding algorithms for defining comorbidities in ICD-9-CM and ICD-10 administrative data. Med Care.

[21] Quan H, Li B, Couris CM, Fushimi K, Graham P, Hider P (2011). Updating and validating the Charlson comorbidity index and score for risk adjustment in hospital discharge abstracts using data from 6 countries. Am J Epidemiol.

[22] Sanders DB, Wolfe GI, Benatar M, Evoli A, Gilhus NE, Illa I (2016). International consensus guidance for management of myasthenia gravis: Executive summary. Neurology.

[23] Mantegazza R, Antozzi C (2018). When myasthenia gravis is deemed refractory: clinical signposts and treatment strategies. Ther Adv Neurol Disord.

[24] Jacob S, Viegas S, Lashley D, Hilton-Jones D (2009). Myasthenia gravis and other neuromuscular junction disorders. Pract Neurol.

[25] Herrett E, Gallagher AM, Bhaskaran K, Forbes H, Mathur R, van Staa T (2015). Data resource profile: Clinical Practice Research Datalink (CPRD). Int J Epidemiol.

[26] NHS Digital. Hospital Episode Statistics (HES). https://digital.nhs.uk/data-and-information/data-tools-and-services/data-services/hospital-episode-statistics#about-the-hes-database Accessed 27 Jan 2020.

[27] Gilhus NE, Verschuuren JJ (2015). Myasthenia gravis: subgroup classification and therapeutic strategies. Lancet Neurol.

[28] Keesey JC (2004). Clinical evaluation and management of myasthenia gravis. Muscle Nerve.

[29] Silvestri NJ, Wolfe GI (2014). Treatment-refractory myasthenia gravis. J Clin Neuromuscul Dis.

