# Supplementary material for: A retrospective longitudinal cohort study of the clinical burden in myasthenia gravis
Source: BMC Neurol. 2022 May 9;22:172. doi: 10.1186/s12883-022-02692-4 (PMC9082838; doi:10.1186/s12883-022-02692-4)
Supplement: Supplementary file 1 — Additional file 1. [file 12883_2022_2692_MOESM1_ESM.docx]

**Additional file 1: Diagnosis codes**

**Supplementary Table 1** Diagnosis codes for myasthenic crises

| **Code type** | **Code** | **Description** |
| --- | --- | --- |
| ICD-10 | J96.00 | Acute respiratory failure |
|  | J96.2 | Acute and chronic respiratory failure |
|  | J96.20 | Acute and chronic respiratory failure, unspecified whether with hypoxia or hypercapnia |
|  | J96.90 | Respiratory failure |
|  | J96.91 | Respiratory failure, unspecified with hypoxia |
|  | J96.92 | Respiratory failure, unspecified, unspecified whether with hypercapnia |
|  | J96.0 | Acute respiratory failure |
|  | J96.9 | Respiratory failure, unspecified |
| Read code | H590.00 | Acute respiratory failure |
|  | R2y1.00 | [D]Respiratory failure |
|  | R2y1x00 | [D]Respiratory failure NOS |
|  | 7L1R.00 | Intubation of trachea |
|  | 7458000 | Invasive ventilation |
|  | 7458100 | Non-invasive ventilation |
|  | 7458300 | Bag valve mask ventilation |
|  | 7458400 | Nebuliser ventilation |
|  | 7459300 | Control of respiration |
|  | 7458y00 | Other specified ventilation support |
|  | 7458z00 | Ventilation support NOS |
|  | 7459y00 | Other specified other respiratory support |
|  | 7459z00 | Other respiratory support NOS |
|  | 87...00 | Respiratory procedures |
|  | 87...11 | Resuscitation - resp. |
|  | 871..00 | Endotracheal intubation |
|  | 871Z.00 | Endotracheal intubation NOS |
|  | 872..00 | Mechanical assistance to resp. |
|  | 872..11 | Intermittent positive pressure ventilation. |
|  | 872..12 | Ventilation-mechanical |
|  | 872Z.00 | Mechanical resp. assist. NOS |
|  | 873..00 | Other resp. resuscitation |
|  | 873Z.00 | Other resuscitation method NOS |
|  | 8H12.00 | Admit to respiratory ITU |
|  | 9NW0.00 | Seen by rapid response team - respiratory |
|  | H585.11 | Adult respiratory distress syndrome |
|  | H585300 | Adult respiratory distress syndrome |
|  | H59..00 | Respiratory failure |
|  | H590.00 | Acute respiratory failure |
|  | R060600 | [D]Respiratory distress |
|  | R060700 | [D]Respiratory insufficiency |
|  | 2324.00 | O/E - respiratory distress |
|  | 7459.00 | Other respiratory support |
|  | 8766.00 | Prescription of respiratory disease rescue medication |
|  | 17Z..00 | Respiratory symptoms NOS |
|  | 17ZZ.00 | Respiratory symptom NOS |
| OPCS Procedure Code | G21.4 | Intubation of oesophagus NEC |
|  | X56.1 | Nasotracheal intubation |
|  | X56.2 | Endotracheal intubation |
|  | X56.8 | Other specified intubation of trachea |
|  | X56.9 | Unspecified intubation of trachea |
|  | E85.1 | Invasive ventilation |
|  | E85.2 | Non-invasive ventilation |
|  | E85.3 | Improving efficiency of ventilation |
|  | E85.9 | Unspecified ventilation support |
|  | G21.5 | Insertion of stent into oesophagus |
|  | G21.8 | Other specified operations on the oesophagus |
|  | X56.3 | Tracheal intubation using laryngoscope |
|  | G21.4 | Intubation of oesophagus NEC |

*ICD-10* International Statistical Classification of Diseases and Related Health Problems 10th revision, *ITU* intensive treatment unit, *NEC* not elsewhere classified, *NOS* not otherwise specified, *OPCS* Office of Population Censuses and Surveys.

**Supplementary Table 2** Diagnosis codes for myasthenia gravis exacerbations

| **Event** | **Code type** | **Code** | **Description** |
| --- | --- | --- | --- |
| MG exacerbation | ICD-10 | G70.01 | Myasthenia gravis acute exacerbation |
|  | Read code | R072.00 | [D]Dysphagia |
|  |  | R072z00 | [D]Dysphagia NOS |
| Intravenous immunoglobulin administration | Read Code | 65O..11 | Immunoglobulin injection |
|  |  | 65O1.00 | Normal immunoglobulin given |
|  |  | 7L10700 | Continuous infusion of immunoglobulin |
|  |  | 7Q0F000 | Immunoglobulins Band 1 |
|  |  | 7Q0F100 | Allergen immunotherapy drugs Band 1 |
|  | OPCS Procedure Code | X96.1 | Immunoglobulins Band 1 |
|  |  | X96.2 | Allergen immunoglobulins |
|  |  | X30.1 | Injection of Rh immune globulin |
|  |  | X30.3 | Injection of immune serum NEC |
|  |  | X35.3 | Intravenous immunotherapy |
|  |  | X37.4 | Intramuscular immunotherapy |
|  |  | X38.5 | Subcutaneous immunotherapy |
|  |  | X89.3 | Immunomodulating drugs Band 1 |
|  |  | X89.8 | Other specified high cost immunosuppressant drugs |
|  |  | X95.1 | Immune response drugs Band 1 |
|  |  | X96.8 | Other specified high cost immunology drugs |
|  |  | X96.9 | Unspecified high cost immunology drugs |
| Plasmapheresis | Read code | 7L12300 | Exchange of plasma (2-9) |
|  |  | 7L13100 | Exchange of plasma (single) |
|  |  | 7L13300 | Exchange of plasma (10-19) |
|  |  | 7L13400 | Exchange of plasma (>19) |
|  |  | 7L15100 | Transfusion of plasma |
|  |  | 7L15600 | Plasmapheresis |
|  |  | 7L15700 | Plasma exchange |
|  |  | 7L15800 | Transfusion of plasma NEC |
|  | OPCS Procedure Code | X32.2 | Exchange of plasma (single) |
|  |  | X32.3 | Exchange of plasma (2-9) |
|  |  | X32.4 | Exchange of plasma (10-19) |
|  |  | X32.5 | Exchange of plasma (> 19) |
|  |  | X34.2 | Transfusion of plasma NEC |
|  |  | X32.8 | Other specified exchange blood transfusion |
|  |  | X32.9 | Unspecified exchange blood transfusion |
| Myasthenic crisis | See Supplementary Table 1 |  |  |

*ICD-10* International Statistical Classification of Diseases and Related Health Problems 10th revision, *ITU* intensive treatment unit, *NEC* not elsewhere classified, *NOS* not otherwise specified, *OPCS* Office of Population Censuses and Surveys.

**Supplementary Table 3** Diagnosis codes for myasthenia gravis-related hospitalization

| **Code type** | **Code** | **Description** |
| --- | --- | --- |
| ICD-10 | G70.00 | Myasthenia gravis without acute exacerbation |
|  | G70.01 | Myasthenia gravis acute exacerbation |
|  | G70.0 | Myasthenia gravis |

*ICD-10* International Statistical Classification of Diseases and Related Health Problems 10th revision.
